# Fetal Programming of Body Composition, Obesity, and Metabolic Function: The Role of Intrauterine Stress and Stress Biology

**DOI:** 10.1155/2012/632548

**Published:** 2012-05-10

**Authors:** Sonja Entringer, Claudia Buss, James M. Swanson, Dan M. Cooper, Deborah A. Wing, Feizal Waffarn, Pathik D. Wadhwa

**Affiliations:** ^1^Department of Pediatrics, School of Medicine, University of California, Irvine, CA 92697-4260, USA; ^2^UC Irvine Development, Health and Disease Research Program, School of Medicine, University of California, Irvine, CA 92697-4260, USA; ^3^Department of Obstetrics & Gynecology, School of Medicine, University of California, Irvine, CA 92697-4260, USA; ^4^Department of Psychiatry & Human Behavior, School of Medicine, University of California, Irvine, CA 92697-4260, USA; ^5^Department of Epidemiology, School of Medicine, University of California, Irvine, CA 92697-4260, USA

## Abstract

Epidemiological, clinical, physiological, cellular, and molecular evidence suggests that the origins of obesity and metabolic dysfunction can be traced back to intrauterine life and supports an important role for maternal nutrition prior to and during gestation in fetal programming. The elucidation of underlying mechanisms is an area of interest and intense investigation. In this perspectives paper we propose that in addition to maternal nutrition-related processes it may be important to concurrently consider the potential role of intrauterine stress and stress biology. We frame our arguments in the larger context of an evolutionary-developmental perspective that supports roles for both nutrition and stress as key environmental conditions driving natural selection and developmental plasticity. We suggest that intrauterine stress exposure may interact with the nutritional milieu, and that stress biology may represent an underlying mechanism mediating the effects of diverse intrauterine perturbations, including but not limited to maternal nutritional insults (undernutrition and overnutrition), on brain and peripheral targets of programming of body composition, energy balance homeostasis, and metabolic function. We discuss putative maternal-placental-fetal endocrine and immune/inflammatory candidate mechanisms that may underlie the long-term effects of intrauterine stress. We conclude with a commentary of the implications for future research and clinical practice.

## 1. Introduction

A growing body of empirical evidence suggests that the origins of obesity and metabolic dysfunction can be traced back to the intrauterine period of life, at which time the developing fetus is acted upon by and responds to suboptimal conditions during critical periods of cellular proliferation, differentiation, and maturation by producing structural and functional changes in cells, tissues and organ systems. These changes, in turn, may have long-term consequences to increase the individual's risk for developing a range of complex common disorders including, but not limited to, obesity and metabolic dysfunction (i.e., the concept of fetal programming of health and disease risk [1]). A large number of  human and animal studies of fetal programming of obesity and metabolic dysfunction have focused on the critical role of maternal nutrition prior to or during gestation and have produced important findings and insights (reviewed in [2–5]). Questions currently under investigation in this context include those related to mechanisms or pathways by which nutritional programming can exert life-long effects on the developing organism. Some of the major nutrition-related pathways discussed in the current literature relate to the effects of maternal nutritional insults on maternal-placental-fetal glucose/insulin physiology and their downstream effects on the developing brain and peripheral systems in the fetal compartment. In this perspectives paper, we argue that it may be important to also simultaneously consider the potential role of intrauterine stress and stress biology for the following reasons: (a) from an evolutionary-developmental perspective, energy availability (i.e., nutrition) and challenges that have the potential to impact the structural or functional integrity and survival of the organism (i.e., stress) represent the most important environmental conditions underlying natural selection and developmental plasticity along all time scales. It is therefore likely and plausible that stress represents an important aspect of the intrauterine environment that would be expected to influence many, if not all, developmental outcomes. (b) stress-related biological factors may exert direct effects on fetal targets of programming of body composition and metabolic function. (c) many of the effects of nutritional insults (both undernutrition and overnutrition) may be mediated by common stress-related pathways involving the hypothalamic-pituitary-adrenal (HPA) axis and its end product, cortisol. Hence, stress biology may represent a common underlying mechanism. (d) stress and stress-related biological processes are known to alter nutrition at several levels, including caloric intake, selection of food types, and metabolic fate of energy. Conversely, nutritional status is also known to alter stress at multiple levels in the brain and periphery, including appraisals of potentially stressful circumstances, psychological and physiological stress responses, and feedback regulation. Hence, in natural settings it is likely that the effects of either nutrition or stress are modified by or conditioned upon the state of the other. In other words, interaction effects, and not main effects, are the more likely scenario underlying causation in the context of complex common disorders including obesity and metabolic function. This issue is particularly important in the human context since nutritional insults and stress tend to cooccur in populations across the world.

 For these reasons, we highlight and review below the effects of stress and stress biology on fetal programming of body composition, obesity, and metabolic function. We review empirical evidence for interactive effects between stress and nutrition, describe findings from some of our own recent studies on prenatal stress and stress biology, and discuss putative maternal-placental-fetal endocrine and immune/inflammatory candidate mechanisms that may underlie and mediate short- and long-term effects of prenatal stress on the developing human embryo and fetus, with a specific focus on body composition, metabolic function, and obesity risk. We conclude with a commentary of the implications for research and clinical practice.

## 2. Rationale for Considering a Role for Stress in Fetal Programming

The origins of health and disease susceptibility for many of the complex, common disorders that confer the major, global burden of disease in developed societies as well as other societies in rapid transition can be traced back to the intrauterine period of life. Development is a plastic process, wherein a range of different phenotypes can be expressed from a given genotype (contained within the fertilized zygote). The unfolding of all developmental processes across the multicontoured landscape from genotype to phenotype is context-dependent, wherein the developing embryo/fetus responds to, or is acted upon by, conditions in the internal or external environment during sensitive periods of cellular proliferation, differentiation and maturation, resulting in structural and functional changes in cells, tissues, and organ systems. These changes may, in turn, either independently or through interactions with subsequent developmental processes and environments, have short- and/or long-term consequences for health and disease susceptibility. These concepts have variously been referred to as the fetal or developmental origins of health and disease risk [1, 6].

The rationale for considering a role for stress and stress biology in fetal programming of child and adult obesity and metabolic dysfunction derives, in part, from concepts in evolutionary biology and developmental plasticity. From conception onwards the mother and her developing fetus both play an obligatory, active role in all aspects of development. Based on the consideration that key environmental conditions that have shaped evolutionary selection and developmental plasticity include not only variation in energy substrate availability (i.e., nutrition) but also challenges that have the potential to impact the structural or functional integrity and survival of the organism (i.e., stress), it is likely and plausible that prenatal stress represents an important aspect of the intrauterine environment that would be expected to influence many, if not all, developmental outcomes [7]. Moreover, we submit the application of a prenatal stress and stress biology framework offers an excellent model system for the study of intrauterine development and associated developmental, birth and subsequent health-related phenotypes because it is increasingly apparent that the developing fetus acquires and incorporates information about the nature of its environment in part via the same biological systems that in an already-developed individual mediate adaptation and central and peripheral responses to endogenous and exogenous stress (i.e., the maternal-placental-fetal neuroendocrine and immune systems [8]).

 Another compelling rationale for considering a role for *in utero* stress as a contributor to subsequent risk of obesity and metabolic dysfunction derives from the effort to elucidate and better understand the underlying reason(s) for the well-documented, persistent and large socioeconomic and racial/ethnic disparities in the population distribution of these outcomes in the US and other developed nations. Many of the factors that disproportionally affect socially disadvantaged individuals, such as medical care, diet/nutrition, and health-related behaviors, have been shown to play only a limited role in accounting for these disparities [9–12]. The search for alternate explanations has led to the hypothesis that high levels of stress may, in part, independently, or in combination with other factors, account for these disparities, because the experience of social disadvantage and minority racial/ethnic status is characterized by higher levels of psychosocial stress and lack of resources, and because stress and stress-related biological processes have been implicated in a wide array of adverse reproductive, developmental, and other health outcomes [9, 13].

 A large body of the literature supports the notion that conditions in the early postnatal period of life (e.g., behavioral or nutritional stress) can induce changes in the metabolic, endocrine, cardiovascular, and behavioral phenotypes, and these effects could be independent of prenatal exposures or could moderate or mediate the effects of prenatal adversity. Several of these findings are derived from animal (rodent) models, in which maturational status over the first two weeks of postnatal life is approximately equivalent to that of the human during the third trimester of gestation. A comprehensive summary of the effects of early postnatal life stress is beyond the scope of the current paper, but we refer to reader to excellent recent reviews on this topic [14, 15].

## 3. The Role of Context: Potential Interactive Effects between Stress and Nutrition

Obesity and metabolic dysfunction are complex, multifactorial outcomes. At the individual level, the major risk categories include sociodemographic, nutritional, historical, biophysical, obstetric, behavioral, psychosocial, genetic, and other environmental factors. Studies of the effects of stress and stress-related processes on these outcomes generally treat other risk factors as potential confounding variables and attempt to account (adjust) for their putative effects by either study design (subject selection criteria) or statistical adjustment. However, emerging concepts of causation for complex common disorders, including but not limited to obesity and metabolic dysfunction, suggest it is not only possible, but in fact probable, that causation does not reside in any single factor or in the additive effects of numerous factors, but lies at the interface between multiple risk factors (interaction or multiplicative [16]). We consider here, by way of example and illustration of this critically important concept, the potential interactive effects between stress and nutrition.

 As discussed briefly in the preceding section, the two fundamental processes that are believed to shape evolutionary selection and developmental plasticity are variation in energy substrate availability (nutrition) and challenges that have the potential to impact the structural or functional integrity and survival of the organism (stress). Maternal nutrition, assessed by indicators of body size such as body mass index (BMI), nutritional intake or serum measures of nutritional biomarkers, is a well-established risk factor for childhood and adult obesity and metabolic dysfunction. Growing evidence supports the concept of a bidirectional interaction between nutrition and stress, such that the effects of nutrition on health may vary as a function of stress, or that the effects of stress on health may vary as a function of nutritional status. For example, several experimental studies in animals have demonstrated that nutritional manipulations, particularly in the preconception or early pregnancy period, may produce their effects on maternal and fetal outcomes via alterations in stress biology (cortisol, inflammatory cytokines [17–25]). Conversely, studies in animals and humans of stress induction (by exposure to laboratory-based stressors or endocrine stress analogues) have demonstrated effects on feeding behavior, food choice (high-calorie dense food preference) and the metabolic fate of food in target tissues [26–30]. For example, chronic stress or cortisol administration motivates people to select high-fat food and to overeat [26, 29], and corticotrophin-releasing hormone (CRH) infusion in healthy human adults also increases subsequent food intake [28]. Furthermore, chronic stress has the potential to impair sleep, and short sleep duration is a predictor of weight gain [31]. In addition, cortisol increases insulin levels [32, 33]. Although insulin is anabolic and under normal basal conditions can increase both lean and fat mass, coelevation of insulin with cortisol preferentially increases abdominal fat stores [34, 35]. Further evidence of an interaction between stress and nutrition comes from a recent study in humans demonstrating that under conditions of stress the brain's energy need increases, and it actively “demands” energy from the periphery (a concept termed “brain-pull,” [36]). It is hypothesized that under conditions of high energy demand the brain can activate its stress systems, that is, the sympathetic nervous system (SNS) and the hypothalamus pituitary adrenal (HPA) axis. Once stress networks in the upper brain stem including the ventromedial hypothalamus (VMH) and the paraventricular nucleus (PVN) are activated, energy—particularly glucose—is allocated to the brain. With SNS activation, insulin secretion from the beta cells is suppressed, and the insulin-dependent glucose uptake via GLUT4 into the body periphery becomes limited, referred to as “cerebral insulin suppression” (CIS). As a consequence of CIS, glucose is available via insulin-independent GLUT1-transport across the blood-brain barrier. This hypothesis was tested using an experimental design, wherein healthy young adults underwent a laboratory stress test and a control session. Acute stress exposure increased carbohydrate intake from a rich buffet compared to the control session. While these stress-extra carbohydrates increased blood glucose concentrations, they did not increase serum insulin concentrations. The ability to suppress insulin secretion was found to be linked to the sympathoadrenal stress response [27]. The authors speculated that disturbances of this “brain-pull” mechanism may be related to the onset of obesity, because in the case of incompetent “brain-pull” food intake has to be increased in order to ensure the brain's energy supply under conditions of stress [36].

 We note that only a small number of studies have examined the relationship between maternal stress and diet or nutritional state in pregnancy. A study by Hurley et al. [37] found that pregnant women who were more fatigued, stressed, and anxious in mid-pregnancy consumed more food (increased macronutrient intake) but concurrently decreased their intake of some micronutrients. Another recent study demonstrated that the level of maternal stress during pregnancy was positively associated with prepregnancy BMI [38]. In an animal model, the interactive effects of maternal stress and nutrition on the subsequent risk of offspring obesity were investigated [24]. Pregnant rats were maintained on standard or high-fat diet throughout gestation and lactation. Offspring from dams that experienced prenatal stress and/or were on a high-fat diet weighed more beginning on postnatal day 7 compared to standard control pups. Access to high-fat diet at weaning increased the body weight effect through early adulthood and was attributable to greater adiposity. Furthermore, pups weaned on to a high-fat diet had impaired glucose tolerance if their dams were on a high-fat diet, experienced prenatal stress, or both [24].

 Findings from a recent study on maternal high-fat diet during pregnancy suggest that the effects on offspring hypertension in adult life are mediated through an exacerbated sympathetic tone that arises very early in life [39]. We note that increased sympathetic tone is also associated with alterations in the stress response.

 Despite the plausibility of stress-nutrition interaction effects in the context of pregnancy, we are not aware of any human studies to date that have examined these interactive effects during pregnancy on offspring body composition and metabolic function.

## 4. Stress-Related Maternal-Placental-Fetal Endocrine and Immune Processes as Potential Mediators of Fetal Programming of Health and Disease

The fetal programming hypothesis has led to the search for underlying mechanisms by which disparate intrauterine insults exert a multitude of effects on different physiological systems in the developing offspring. A question of particular interest relates to whether these biological mechanisms are exposure and/or outcome-specific, or whether there may be some common mechanisms that mediate the effects of various exposures on a range of disparate outcomes. We suggest that stress-related maternal-placental-fetal endocrine and immune processes in gestation constitute an attractive underlying common candidate mechanism because they are responsive to many classes of intrauterine perturbations and they act on multiple targets of fetal programming [8, 40]. Unlike exposure to toxins and teratogens, it is important to appreciate the fact that maternal-placental-fetal hormones and cytokines play an essential and obligatory role in orchestrating key events underlying cellular growth, replication and differentiation in the brain and peripheral tissues [41–46]. Thus, perturbations in the level and/or time of exposure of these biologic effectors are likely to produce alterations of normal structure and function. It is also important to appreciate that the state of pregnancy itself produces major and progressive alterations in the function of these systems, and that these changes may have important implications for altering the responsivity of these systems to exogenous or endogenous perturbations and hence their downstream effects on fetal targets of programming.

### 4.1. Stress Biology in Human Pregnancy

Stress biology refers to the set of biological adaptations in response to challenges or demands that threaten or are perceived to have the potential to threaten the stability of the internal milieu of the organism. The nervous, endocrine, immune, and vascular systems play a major role in adaptations to stress. There are no direct neural, vascular, or other connections between the mother and her developing fetus—all communication between the maternal and fetal compartments is mediated via the placenta, an organ of fetal origin. Based on the physiology of stress, parturition and the evidence linking maternal stress to earlier delivery, we have previously proposed a biobehavioral framework of stress and adverse birth outcomes [8], that may also be applicable in the present context.

Pregnancy produces major alterations in neuroendocrine and immune function, including changes in hormone and cytokine levels and control mechanisms (feedback loops), that are crucial in providing a favorable environment within the uterus and fetal compartment for growth, differentiation and maturation, and conveying signals when the fetus is ready for extrauterine life. Starting very early in gestation the placenta, the first fetal organ to develop and function, produces hormones, neuropeptides, growth factors, and cytokines, and appears to function in a manner resembling that of compressed hypothalamic-pituitary-target systems [47].

#### 4.1.1. Maternal-Placental-Fetal Stress-Related Endocrine Function

Glucocorticoid physiology (cortisol in humans) has received extensive and well-placed consideration as a critical endocrine mediator of fetal programming, with an emphasis on not only hormone production but also hormone action mediated by tissue-specific glucocorticoid receptor expression, sensitivity and affinity, and by maternal-fetal transfer mediated by the activity of the placental 11*β*-hydroxysteroid dehydrogenase enzyme system (see [48] for a recent review). Less well recognized is the potential and perhaps equally important role of the peptide corticotrophin-releasing hormone (CRH). In primates, but not other mammals, the placenta synthesizes and releases CRH in large amounts into the fetal and maternal circulations. In contrast to the inhibitory influence on hypothalamic CRH production, cortisol stimulates placental CRH production [49], and this positive feedback loop results in a progressive amplification of CRH and cortisol production over the course of gestation [50].

#### 4.1.2. Maternal-Placental-Fetal Stress-Related Immune Function

With respect to the immune axis, a major endeavor of pregnancy-related alterations in immune function is to achieve and maintain the optimal balance between tolerating the fetal semiallograft while not suppressing maternal immune responses to an extent that increases maternal or fetal susceptibility to infection. Thus, a generalized reduction of maternal immune responsiveness occurs during pregnancy, mediated by hormonal changes (e.g., increased levels of progesterone), trophoblast expression of key immunomodulatory molecules, and a progressive switch from a TH*_1_*/TH*_2_* balance to a predominantly T-helper 2-type pattern of cytokines [51].

#### 4.1.3. Interactions between Maternal-Placental-Fetal Neuroendocrine, Immune-Inflammatory, and Vascular Pathways in Pregnancy

Although distinct neuroendocrine, immune/inflammatory, and vascular pathways have been described, growing evidence suggests that these and other physiological systems involved in pregnancy are highly interrelated, and that they extensively regulate and counterregulate one another. The potential complexity of the interrelationships among these physiologic systems is seen when considering the role of infection in the etiology of adverse fetal developmental and birth outcomes. For example, inflammatory cytokines that are produced in response to infection, such as TNF-*α*, IL-1*β*, and IL-6, can activate components of the MPF neuroendocrine system [52–54]. Conversely, it is also known that HPA hormones such as CRH and cortisol influence the production of cytokines and modulate the inflammatory response to infection [55–57]. Central CRH, acting via glucocorticoids and catecholamines, inhibits inflammation, whereas CRH directly secreted by peripheral nerves and mast cells stimulates local inflammation [58]. Impaired nutrient and oxygen exchange associated with uteroplacental vasculopathy may stress the fetus and result in increased production of placental-fetal hormones such as CRH, while placental CRH, in turn, may influence fetal-placental circulation [59]. Thus, the relationship between immune and endocrine alterations during pregnancy to adverse metabolic outcomes and increased risk of obesity on the offspring is likely to involve complex interactions between the endocrine, immune, and vascular systems.

### 4.2. Prenatal Stress and Maternal-Placental-Fetal Endocrine and Immune Function

Substantial evidence in nonpregnant humans and animals demonstrates that stress exposure produces activation of the neuroendocrine system (e.g., HPA axis) and exaggerated inflammatory responses [60, 61]; however, these associations cannot be assumed to also be present in the pregnant state because the above-described changes in endocrine and immune physiology have consequences for attenuating the responsivity of these systems to stress. With respect to prenatal psychosocial stress-related biological pathways, some of our earlier studies were among the first to demonstrate that despite the large pregnancy-associated changes in maternal endocrine physiology, the system is responsive to maternal psychosocial states (such as high stress and low social support) [62]. Our more recent studies on maternal stress responses in human pregnancy are among the first to demonstrate that maternal psychophysiological stress responses are progressively attenuated with advancing gestation [63], and that after accounting for the effects of other established risk factors, the degree of attenuation is a significant predictor of shortened length of gestation and earlier delivery [64].

Studies by other groups have reported that elevated psychosocial stress in pregnant women is associated with higher circulating levels of inflammatory markers like C-reactive protein (CRP) and the proinflammatory cytokines IL-1b, IL-6, and TNF-*α*, with lower circulating levels of the anti-inflammatory cytokine IL-10 and *ex vivo* endotoxin (LPS)-stimulated levels of IL-1b and IL-6 [65, 66]. Another recent study of pro-inflammatory responses to an *in vivo* antigen challenge (influenza virus vaccination) in pregnant women reported an association between depressive symptoms and sensitization of the inflammatory cytokine responses [66].

 In addition to psychosocial stress, substantial *in vitro* and *in vivo* evidence indicates that maternal-placental-fetal endocrine and immune processes during pregnancy respond to a variety of other maternal and intrauterine perturbations, including biological effectors of stress [67–72], obstetric risk conditions such as preeclampsia, pregnancy-induced hypertension [57–70], gestational diabetes [73, 74], infection [75–78], reduced uteroplacental blood flow [79, 80], and behavioral factors such as the constituents of maternal diet, over- and under-nutrition, and smoking [17–23, 81, 82].

Based on these findings, it is apparent that measures of maternal-fetal endocrine and immune/inflammatory stress markers capture physiological responses to a wide range of intrauterine perturbations including, but not limited to prenatal stress. In accordance with our suggestion that stress-related maternal-placental-fetal endocrine and immune processes in gestation constitute an attractive candidate mechanism for fetal programming, a recent *JAMA* editorial [83] on an article reporting an increase in the prevalence of several categories of chronic illness in childhood, including obesity, asthma, and ADHD [84], speculates that there may be common early risks underlying these conditions that are triggering development of aberrant physiologic pathways. The editorial suggests that adverse early experiences that affect stress-sensitive physiologic systems (endocrine/metabolic, immune) may contribute to not only the onset of childhood illness but also predispose the same individuals to develop age-related diseases as adults.

## 5. Long-Term Effects of Prenatal Stress Exposure on Human Adult Physiology and Health

The majority of human epidemiologic studies of the fetal programming hypothesis have operationalized unfavorable intrauterine environments using indicators of adverse birth outcomes such as low birth weight. We and others have argued that the long-term effects on child or adult disease-related phenotypes of interest may not necessarily be mediated by adverse birth outcomes. For example, several experimental studies in animals suggest that maternal exposure to psychosocial stress during gestation can independently exert long-term effects on several central and peripheral systems in the offspring, and that titration of the prenatal stress exposure dose can produce significant long-term effects without necessarily altering the birth phenotype [85–89]. However, only a very small number of studies have investigated this issue in humans. As a first step to addressing this question, we conducted a study using a retrospective case-control design in a sample of healthy young adults born to mothers with healthy pregnancies and normal birth outcomes. One half of the study population of young adults was born to mothers who had experienced a major stressful life event during the index pregnancy (prenatal stress group (PS)), whereas the other half was a sociodemographically matched population with no history of maternal exposure to prenatal stress (comparison group (CG)). We selected a study population of younger as opposed to older adults in order to focus on predisease markers of physiological dysregulation of metabolic, endocrine, and immune systems as early predictors of disease susceptibility. The potential effects of other established obstetric, newborn, and childhood risk factors on adult health were controlled using a stringent set of exclusionary criteria. Maternal and child medical records were obtained and screened to exclude presence of any maternal acute or chronic diseases, obstetric complications (e.g., gestational diabetes, hypertension/preeclampsia, and infection), unhealthy behaviors (smoking), adverse birth outcomes (preterm birth, low birth weight), newborn complications, and history of any major childhood or current diseases (obesity, diabetes, asthma, and adverse neurodevelopmental or psychiatric conditions). Study assessments were performed to quantify health and physiological markers of disease risk, including (i) body composition and glucose-insulin metabolism (BMI and percent fat mass; basal and postoral glucose tolerance test levels of glucose, insulin, leptin, adiponectin; fasting lipid profile), (ii) endocrine function (basal and post behavioral/pharmacological stress levels of pituitary-adrenal stress hormones, chronobiological regulation of adrenal function, and assessment of HPA-axis feedback sensitivity), (iii) immune function (immune cell trafficking and phytohemagglutinin (PHA)-stimulated production of pro- and anti-inflammatory and TH_1_/TH_2_ cytokines), (iv) cognitive function (working memory under basal and hydrocortisone conditions), and (v) cellular aging (as indexed by leukocyte telomere length). Because subtle physiological differences in disease susceptibility are often not detected in basal state, we employed appropriate challenge tests to quantify the function of these systems under stimulated conditions (e.g., oral glucose challenge, ACTH stimulation test, PHA-stimulated immune responses, and working memory after cortisol administration).

 Our results (summarized in Table 1) indicated that the young adults exposed during intrauterine life to maternal psychosocial stress consistently exhibited significant dysregulation of all these key physiological parameters, thereby placing them at increased risk for developing complex common disorders. Specifically, individuals in the PS group exhibited higher BMI and percent body fat, primary insulin resistance, and a lipid profile consistent with the metabolic syndrome [90]; altered immune function with a TH2 shift in the TH1/TH2 balance (consistent with increased risk of asthma and autoimmune disorders [92]); altered endocrine function, with an increased ACTH and reduced cortisol levels during pharmacological and psychological stimulation paradigms (consistent with the high-risk endocrine profile exhibited by individuals exposed to early life abuse [95]); accelerated cellular aging (as indexed by shortened leukocyte telomere length that extrapolated to approximately a 3.5-year increase in the rate of cell aging [82]); and impaired prefrontal cortex (PFC)-related cognitive performance (impairments in working memory performance after hydrocortisone administration) [93]. Interestingly, stress-related changes in PFC function are believed to play a role in alterations of hypothalamic energy balance homeostasis circuits and obesity risk (see, for example, [96, 97]) thereby suggesting that prenatal stress may program brain regions that are associated with the control of energy intake. Consistent with the finding on cognitive function are results from one of our other recent prospective, longitudinal studies on the long-term effects of prenatal stress (anxiety) on child brain morphology. After excluding cases with low birth weight and adjusting for total gray matter volume, age, gestational age at birth, handedness, and postpartum stress, maternal pregnancy-specific anxiety in mid-gestation was associated with gray matter volume reductions in several child brain regions, including the prefrontal cortex [98].

Taken together, our findings suggest that *in utero *exposure to prenatal psychosocial stress may confer increased long-term risk of a range of negative physiological and cognitive health outcomes in humans; these effects are independent from those of other established obstetric and childhood risk factors; these long-term effects are not necessarily mediated by unfavorable birth outcomes. It is noteworthy that our above-described finding on body composition is consistent with a more recent report in a large, national cohort sample linking prepregnancy and prenatal stress exposure related to maternal bereavement to risk of childhood overweight [99], and our finding on immune function is consistent with another recent report linking prenatal maternal anxiety with infant illnesses and antibiotic use [100]. We also note that our findings on prenatal stress-associated altered immune, endocrine, cell aging, and cognitive function all converge in a manner consistent with the programming of body composition, obesity risk, and metabolic dysfunction.

## 6. Fetal Programming of Body Composition, Metabolic Function, and Obesity Risk

Continuing with the theme of a common underlying biological mechanism, in this section we address the issue of the potential impact of intrauterine stress biology on multiple targets of fetal programming related to body composition, metabolic function and obesity risk (see also [40]).

 Obesity (or, to be more precise, adiposity) is recognized as one of the most serious health problems in the US and other societies. At the individual level, obesity results when energy intake exceeds energy expenditure. However, the relationship between excess energy intake and adiposity is not linear and monotonic; there is wide variation among children or adults at identical levels of excess energy intake in their propensity to gain weight and accrue fat mass. This variation across individuals defines susceptibility for developing obesity/adiposity. Once an individual becomes obese, it is difficult to lose weight, and even more difficult to sustain weight loss, because of the remarkable efficiency of energy balance homeostasis mechanisms [101–103]. For these reasons, it is important to gain a better understanding of the origins of individual differences in the propensity for weight and fat mass gain, in order to predict obesity risk and develop strategies for primary prevention [102].

### 6.1. Targets of Programming of Obesity: Potential Role of the Maternal-Placental-Fetal Endocrine and Immune/Inflammatory Pathway

**Figure 1:**
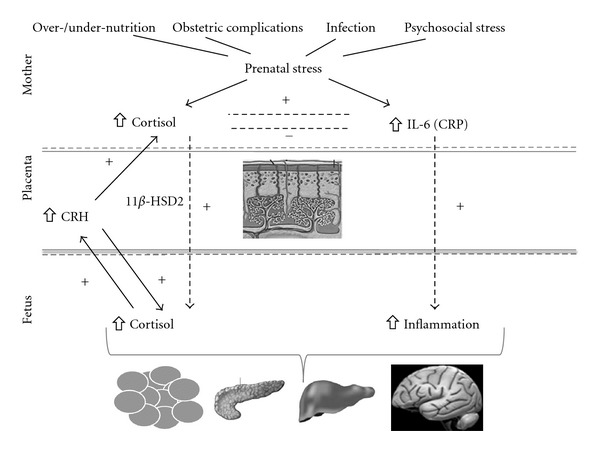
Intrauterine stress biology and programming of fetal targets of body composition and metabolic function. Adverse circumstances during pregnancy (physiological as well as psychological stressors, summarized here as “prenatal stress”) have the potential to induce changes in maternal-placental-fetal stress biology (e.g., increases in maternal and fetal cortisol, placental corticotrophin-releasing-hormone (CRH), and inflammatory mediators). The subsequent increase in stress hormones and proinflammatory cytokines in the fetal compartment during sensitive or critical developmental windows can impact the structure and function of the brain and peripheral targets (e.g., adipose tissue, pancreas, and liver) that are related to body composition, energy balance homeostasis, and metabolic function.

It is well established that the primary targets of programming of body composition, metabolic function, and obesity risk are the neural networks that regulate energy balance (appetite, feeding, and basal energy expenditure) and peripheral organs and tissues involved in fat synthesis/breakdown, storage and metabolic function (adipocyte, liver, pancreas, and muscle). In this section, we consider and review findings that pertain to the potential role of prenatal stress biology in programming these major targets of interest (see Figure 1).

 Stress-related endocrine and immune processes in human pregnancy are associated with not only fetal development and birth outcomes but also with later disease risk. For example, we have reported that placental CRH concentrations in human pregnancy significantly predict the rate of fetal growth and size at birth [104], which, in turn, is a significant predictor of childhood and adult adiposity [105–107]. Other researchers have found a positive association between CRH levels in pregnancy and an increase in central adiposity [108] and alterations in adiponectin levels in 3-year-old children [109]. Yet others have reported a positive association between maternal levels of interleukin-6 (IL-6) in pregnancy and neonatal adiposity [110]. In a recent large epidemiological study in humans, Li et al. [101] found an association between maternal bereavement from death of someone close during pregnancy and an increased risk of overweight in the offspring in later childhood. Furthermore, animal studies have demonstrated long-term effects of prenatal stress exposure on increased body weight in the offspring [24, 111].

#### 6.1.1. Neural Circuits

The central role of ventromedial hypothalamic (VMN) circuits in regulating feeding and energy balance is well established. VMH neurons contain receptors for and receive afferent signals related to fat stores (leptin), nutrient metabolism (insulin), hunger (ghrelin), and satiety (peptide YY), and they integrate peripheral signals of effectors of food intake and energy expenditure so as to prevent substantial variations in the level of energy balance [112]. Also involved in the regulation of appetite and food intake are brain regions that make food intake rewarding (limbic structures), and higher cortical structures (e.g., prefrontal cortex) that are important for learned patterns of eating behavior and executive control [96]. A growing body of the literature suggests that intrauterine perturbations can produce reorganization of these neural pathways that regulate energy intake and expenditure in ways that enhance the development of obesity. Several studies have convincingly demonstrated that biological (endocrine, immune) stress during gestation, triggered by a variety of nutritional, inflammatory, vascular, behavioral, or psychosocial perturbations, can promote obesity in the offspring by reorganizing central neural pathways through programming of energy balance “set points.” (see [113] for recent review). One key system involved in the regulation of energy balance is the hypothalamic (CRH)-pituitary (ACTH)-adrenal (cortisol) neuroendocrine stress axis, which forms a network of neuronal pathways capable of interacting with brain circuits controlling energy balance [114]. For instance, the adipogenic hormone leptin which is the afferent loop informing the hypothalamus about the states of fat stores, participates in the expression of hypothalamic CRH, interacts at the adrenal with ACTH, and is regulated by cortisol. Cortisol increases leptin secretion and limits CNS leptin-induced efferents [115].

#### 6.1.2. Adipocytes

Obesity is impacted by increases in fat cell number, size, or both. Fetal adipose tissue development is regulated by the complex interaction of maternal, endocrine, and paracrine influences that initiate specific changes in angiogenesis, adipogenesis, and metabolism [116]. Adipogenesis, the process of adipocyte development from mesenchymal stem cell precursors, occurs primarily during late fetal and early postnatal life in humans, and the number of adipocytes is relatively fixed after young adulthood [116–118], supporting the notion that fetal and early postnatal periods are crucial windows in the development of adipose depots. Adipogenesis is highly sensitive to the intrauterine biological environment, in particular to concentrations of insulin-like growth factors, glucose, insulin, and glucocorticoids [116, 117]. *In vitro* studies could show that the differentiation of human adipocyte precursor cells in the presence of insulin is stimulated by cortisol in a dose-dependent manner and occur at physiological concentrations [119, 120]. Furthermore, *in vitro* exposure of isolated human adipocytes to insulin and corticosteroids synergistically induces peroxisome proliferator-activated receptor (PPAR-*γ*) mRNA expression [121].

 CRH seems to be an important regulator of adipocyte function, and CRH receptors are expressed in both white and brown adipocytes [122]. The role of cytokines as regulators of adipose tissue metabolism is well established. Proinflammatory cytokines are elevated in obese individuals, and they seem to modulate leptin secretion from adipocytes [123]. Furthermore, in an animal study prenatal exposure to pro-inflammatory cytokines or dexamethasone had an effect on increased fat depots in the offspring [124]. Animal studies have shown that fat cells exposed to an excess substrate supply during crucial windows in their development have an increased capacity for storing lipid in postnatal life [125, 126]. This enhanced lipogenic capacity renders these individuals more likely to store excess energy in the form of fat and increases their susceptibility to weight gain and obesity and its metabolic sequelae. In individuals exposed to low nutrition levels before birth, adipocyte development is initially sacrificed in favor of “essential” organs [4, 127]. If an *in utero* “restricted” individual is born into a postnatal environment in which nutrient supply is no longer constrained, a period of “catchup” fat deposition ensues, mainly in the visceral adipose depot [128]. These individuals are at increased risk of visceral obesity [107] and, consequently, to the development of insulin resistance and type 2 diabetes [129].

#### 6.1.3. Liver and Pancreas

The liver controls the production and fate of metabolic fuels through the action of hepatic enzymes. Phosphoenolpyruvate carboxykinase (PEPCK), a key enzyme in hepatic gluconeogenesis, is under potent glucocorticoid regulation. In animals, prenatal exposure to dexamethasone produces an increased expression of hepatic glucocorticoid receptors as well as increased levels and activity of PEPCK [130], thereby predisposing these animals to glucose intolerance later in life. Furthermore, manipulation of diet during pregnancy is associated with epigenetic changes in the promotor regions of the genes encoding PPAR*α* and the glucocorticoid receptors in the liver in offspring after birth, thereby altering their metabolic phenotype [131, 132]. Insulin is produced by the beta cells in the pancreas in response to elevated blood glucose levels. Increased glucocorticoid exposure and malnutrition during fetal development have the potential to permanently reduce the pancreatic beta cell mass and lower pancreatic insulin content, thereby increasing the risk for metabolic disease later in life (reviewed in [133]). For example in humans, prenatal exposure to glucocorticoids or stress was associated with higher insulin resistance in the adult offspring [90, 134]. 

### 6.2. Genes, Gene-Environment Interactions, and Epigenetic Mechanisms

The ascertainment of genetic contributors to body composition, obesity, and metabolic dysfunction is an area of active and intense investigation. Although weight and body composition are highly heritable, known genes account for only a modest proportion of their variance [135–137]. Genetic makeup alone cannot explain the rapid increase in obesity prevalence in the population because the genetic characteristics of the human population have not changed in the last three decades, but the prevalence of obesity has tripled during that time [138]. Estimates of maternal transmission of heritability are stronger than those for paternal transmission, which argues in favor of intrauterine effects and/or mitochondrial DNA effects. Moreover, the strongest genetic associations seem to vary as a function of the environment (e.g., effects are seen at specific times but not other times in the life cycle). These observations suggest gene-environment interactions are particularly relevant in the context of the obesity phenotype.

The search for mechanisms by which environmental conditions during development can produce long-term changes in the structure and function of cells, tissues, and organ systems has led to the identification and study of epigenetic processes in the context of fetal programming of health and disease risk. A detailed review of epigenetics is beyond the scope of the current paper, and we have elaborated on this issue elsewhere [139, 140]. The effects of nutritional conditions in early development on epigenetic processes have been studied extensively (for recent reviews see, for example, [6, 141–147]).

 We suggest that the incorporation of the genetics and epigenetics of stress and stress biology in the context of fetal programming of body composition, obesity, and metabolic function is likely to yield additional important information that supplements and complements investigations of nutrition-mediated genetic and epigenetic processes underlying fetal programming. As discussed above, many of the effects of maternal nutrition on the developing embryo and fetus may be mediated, in part, by stress hormones such as CRH and cortisol. Diet in prenatal or early postnatal life has been shown to alter the methylation status of several genes implicated in stress and stress physiology, including genes encoding the glucocorticoid receptor and proopiomelanocortin [141]. Conversely, intrauterine or early postnatal exposure to inappropriate levels of stress hormones such as glucocorticoids is known to produce a wide array of epigenetic modifications in tissues including the placenta, brain, adipose tissue, liver, lungs, kidney, skeletal muscle, heart, and blood vessels (for a recent review see [148]). Many of these changes have important long-term implications for body composition and metabolic function. Interestingly, a variant in the gene encoding the glucocorticoid receptor has been associated with increased body fatness in children [149]. We and others have described the association of the same variant with altered physiological stress responses [150]. Furthermore, manipulation of maternal behavior in the early postnatal period in rats permanently alters the offspring's epigenome at the glucocorticoid receptor gene promoter in the hippocampus, and this altered methylation state is associated with changes in GR expression and hypothalamic-pituitary-adrenal (HPA) responses to stress in the offspring [151].

 With respect to the issue of the contribution of stress and stress biology it is very likely that genetic and epigenetic variations will be determined to play an important role in moderating the association between intrauterine stress and obesity and metabolic dysfunction risk via maternal-fetal gene-gene and gene-environment interactions at multiple levels, originating with the likelihood of encountering stressful life circumstances, and culminating in modifying the effects of stress-related biological processes on relevant target tissues. For example, women who are carriers of certain genotypes (high-risk alleles in dopamine-related genes) may be more likely to place themselves in stressful life circumstances [152–154]. The psychological appraisal of potentially stressful circumstances may be influenced directly by the maternal genotypic variation (e.g., in the serotonin transporter gene [155]) or indirectly by the fetal genotype (via its effect on alterations in maternal physiology that, in turn, influence maternal psychological appraisals). Next, the ensuing effects of maternal stressful experience on maternal and fetal biology may be moderated by the genetic and epigenetic characteristics of the mother and fetus (e.g., variants in the glucocorticoid receptor gene [156]), respectively. Finally, the effects of stress-related physiological alterations on target placental and fetal tissues implicated in energy balance homeostasis and metabolic function may be further influenced by the genetic and epigenetic makeup of the mother and fetus, respectively.

 To date, only a small number of studies have systematically addressed the issue of a genetic predisposition for susceptibility to psychosocial stress and related psychobiological states. For example, we and others have described that certain polymorphisms in the promotor region of the gene encoding the glucocorticoid receptor are associated with changes in the regulation of the HPA axis at different levels including basal level, feedback regulation, and response following a psychosocial stressor (summarized in [156]). Because several genes that code for proteins involved in the regulation of the stress response also are involved in the physiology of pregnancy and fetal development (e.g., CRH, cortisol, IL-6, etc.), individual differences in genetic variation may be another factor underlying susceptibility in terms of the potentially adverse effects of maternal stress on pregnancy outcomes. The participation of placental CRH as a central molecule in regulating various aspects of pregnancy, fetal development, and birth outcomes has been discussed in the preceding sections. Thus, DNA sequence variations in the CRH gene, the CRH receptor genes, the glucocorticoid receptor gene, and other genes encoding key enzymes and binding proteins in their biosynthetic pathways may have important implications in this context.

 Genetic and epigenetic mechanisms have been proposed to explain the observed racial/ethnic disparities in obesity and metabolic diseases, particularly with respect to the hypothesized contribution of prenatal stress. These observed racial/ethnic disparities are commonly assumed to reflect the burden of adverse societal conditions associated with minority racial/ethnic status in the US. Prenatal stress is a plausible mediator of the effects of race/ethnicity via one or both of two possibilities: greater cumulative exposure to stress and greater vulnerability to the effects of stress (arising from differences in psychobiological responses to stress). The characterization of racial/ethnic differences in DNA sequence or epigenetic variation in genes associated with the stress response will prove particularly informative in this regard. For instance, we and others have previously reported significant racial/ethnic differences in stress-related hormonal states in human pregnancy [157, 158]. These racial/ethnic differences in neuroendocrine function in pregnancy may, in turn, reflect one or more of three possibilities: first, that particular genetic variations associated with pathophysiology are more frequent in specific racial/ethnic populations. Second, that there are no differences in the frequency of particular genetic variations across population subgroups; however, they are phenotypically expressed only under certain environmental conditions or exposures associated with particular racial/ethnic populations (e.g., high stress, reproductive tract infection, social or cultural behavioral practices). And third, that there are no differences in the frequency of particular genetic variations across population subgroups; however, specific gene regions are preferentially expressed or silenced by epigenetic modifications that occurred during sensitive or critical periods of the mother's own development under environmental conditions associated with particular racial/ethnic populations (e.g., intrauterine exposure to high stress or infection [159]). One of our ongoing projects is in the process of evaluating these possibilities by determining whether the population structure and functional significance of maternal-fetal genetic variation and gene-environment interactions vary as a function of race/ethnicity.

## 7. Future Directions: Implications for Research and Clinical Practice

By incorporating the developmental programming approach into the traditional paradigm of causation of complex common health disorders, the focus shifts to placing a far greater emphasis on the health and well being of young women of reproductive age prior to conception and across gestation, in order to more effectively address health and disease risk-related issues in their offspring from infancy and childhood through adolescence and into adult life. A multilevel approach is required that includes molecular and cellular studies, the use of appropriate animal models, and well-designed human studies. In the context of human research, opportunities are limited for experimental manipulations of prenatal stress and the intrauterine environment, and for access to many of the target tissues of interest, particularly in fetal life. Hence, for future research purposes, the value of prospective, longitudinal, follow-up studies, ideally starting before conception, and extending through pregnancy and birth into childhood and beyond, is emphasized. For these studies, deployment of state-of-the art methods, including the assessment of metabolomic and gene expression profiles to precisely characterize maternal nutritional biomarkers and their interactive effects with stress biology during pregnancy, 3D/4D fetal ultrasonography for quantification of fetal growth (biometry), regional blood flow (uterine, umbilical, and cerebral), hepatic and renal volume [160, 161], growth trajectory of organs (placenta, brain, liver, kidneys, and adrenals [162, 163]) and body composition (arm, thigh, and visceral fat/lean mass [164, 165]), coupled with reliable assessments in newborns, infants and children of body composition (with magnetic resonance imaging (MRI) or dual energy X-ray absorptiometry (DXA)) and energy expenditure (basal metabolic rate and total energy expenditure using indirect calorimetry and the doubly labeled water method (DLW), resp.), will move the field forward in an informed manner. Furthermore, recent advances in imaging techniques will likely enable the developments of protocols in infants and children for subcutaneous and visceral fat quantification (especially intrahepatic fat) [166], and characterization of and differentiation between white and brown adipose tissue [167]. These observational studies, in conjunction with parallel molecular studies including studies of human placental, multipotent (stromal) stem cells, and adipose tissue culture systems [168], and coupled with state-of-the-art statistical modeling techniques for parametric and nonparametric repeated measures, time-series data [169–172], will contribute to further defining technical capabilities in this field.

 Regarding clinical implications, it is apparent that current approaches to the prevention and management of obesity and associated metabolic disorders have yielded only very limited success. Once an individual becomes obese, it is difficult to lose weight, and even more difficult to sustain weight loss [101–103]; systematic studies of the efficacy of current weight loss programs have provide the sobering statistic that approximately 80–90% of obese people who have lost weight regain it within one year [173–175]. Clearly, it is critical to adopt a developmental framework in order to arrive at a better understanding of the origins of individual differences in the propensity for weight and fat mass gain, and to develop and test hypotheses that set the stage for translational research to inform the subsequent development of primary intervention strategies before an individual becomes overweight or obese, or secondary interventions to increase the likelihood of a favorable and more sustained response to weight loss strategies.

## 8. Conclusion

Based on the conceptual framework and empirical findings presented here, we suggest that in addition to maternal nutrition it is important to also consider the potential role of intrauterine stress and stress biology in arriving at a better understanding of developmental programming of health and disease susceptibility. Moreover, we submit that stress-related maternal-placental-fetal endocrine and immune processes in human gestation represent a potentially attractive underlying candidate mechanism for elucidating the common biological basis (pathway) for mediating not only the long-term effects of prenatal stress but also those of a host of other intrauterine perturbations including maternal over- and under-nutrition that have been implicated in this area.

## Figures and Tables

**Table 1:** Long-term effects of prenatal stress exposure in young adults: summary of our studies.

Outcome	Finding	Potential implications	Reference
Body composition and metabolic function	↑ BMI; ↑ % body fat ↑ Insulin 2 h after oral glucose tolerance test ↑ Leptin ↓ Fasting HDL; ↑ fasting VLDL	Risk for cardiometabolic disorders/type 2 diabetes	Entringer et al. 2008 *Am J Ob Gyn* [90]

Endocrine system	↑ ACTH, ↓ cortisol in response to psychosocial stress test ↓ Cortisol levels in response to ACTH_1-24_ stimulation test	Susceptibility for psychosomatic disorders	Entringer et al. 2009 *Horm Behav *[91]

Immune system	TH2 shift in TH1/TH2 balance after PHA stimulation ↑ IL-6, IL-10 after PHA stimulation	Risk for allergies, atopic disease, and asthma	Entringer et al. 2008 *Dev Psychobiol *[92]

Cognitive function	↓Working memory performance after hydrocortisone administration	Impaired prefrontal cortex-related executive function	Entringer et al. 2009 *Behav Neurosci* [93]

Cellular aging	↓ Leukocyte telomere length	Risk for age-related degenerative disorders	Entringer et al. 2011 *PNAS* [94]

BMI: body mass index; HDL: low-density lipoprotein; VLDL: very low-density lipoprotein; ACTH: adrenocorticotrophic hormone; PHA: phytohemagglutinin; TH: T-helper cell; IL: interleukin.
